# Perceptions of Exercise Benefits and Barriers and Physical Activity Status Among Patients Undergoing Hemodialysis in Saudi Arabia

**DOI:** 10.3390/healthcare14050592

**Published:** 2026-02-27

**Authors:** Mohanad Almaimani, Enad Alsolami, Abdullah Hussien Alghamdi, Abdullah Alaryni, Nader Mohamed Habib, Mohammed Hassan Hussain Elamin, Khalid Hamed Alhougail, Abrar Alamrani, Sami Alobaidi, Nada Khalid S. Bashnini

**Affiliations:** 1 Department of Internal Medicine, College of Medicine, University of Jeddah, Jeddah 23218, Saudi Arabia; eaalsolami@uj.edu.sa (E.A.); salobaidi@uj.edu.sa (S.A.); nkbashnini@uj.edu.sa (N.K.S.B.); 2 College of Medicine, Imam Mohammad Ibn Saud Islamic University (IMSIU), Riyadh 11623, Saudi Arabia; dr.alhomrani@gmail.com (A.H.A.); aaalaryni@imamu.edu.sa (A.A.); 3 Abdulkarim Bakr Medical Center, Jeddah 23521, Saudi Arabia; n.habieb@albir.org.sa (N.M.H.); m.alameen@albir.org.sa (M.H.H.E.); 4 Department of Medicine, Dr. Sulaiman Al Habib Medical Group, Riyadh 11372, Saudi Arabia; khalidalhokel@gmail.com (K.H.A.); abrarmarwan97@gmail.com (A.A.)

**Keywords:** barriers, benefits, exercise, hemodialysis, physical activity, Saudi Arabia

## Abstract

**Background:** Physical exercise is a potential non-pharmacological therapy for patients with end-stage renal disease (ESRD). Perception of benefits and barriers of exercise among hemodialysis (HD) patients is linked with their exercise behavior. This study aimed to investigate perceptions of exercise benefits and barriers among patients on HD in Saudi Arabia and their associated predictors. **Methods:** This is a cross-sectional survey study that was conducted in two dialysis centers in Saudi Arabia between May and September 2025. In this research, the Dialysis Patient-perceived Exercise Benefits and Barriers Scale (DPEBBS) was used to examine patients’ perceived benefits and barriers to exercise. Patients’ physical activity status was evaluated using the General Practice Physical Activity Questionnaire (GPPAQ). Multivariate logistic regression analysis was used to define factors influencing positive perception towards physical activities and perceived barriers. **Results:** This study included 104 patients with ESRD. Most patients expressed positive perceptions regarding the benefits of exercise. The majority agreed or strongly agreed that exercise improves mood (87.5%), prevents muscular atrophy (81.8%), postpones decline in body function (81.7%), and enhances quality of life (83.7%). Regarding barriers to exercise, several concerns were frequently reported by patients. The most prominent barrier was worry about affecting the arteriovenous fistula. Tiredness (70.2%) and muscle fatigue (63.5%) were also commonly cited obstacles. Age was significantly associated with lower odds of perceiving exercise as beneficial (aOR = 0.94; 95%CI:0.91–0.98; *p* = 0.008). This indicates that older patients were slightly less likely to report a positive perception towards exercise. Having a bachelor’s degree is associated with higher odds of reporting exercise barriers compared with no education (aOR = 16.22, 95%CI:1.29–204.42; *p* = 0.03). The majority of the patients (78.8%) are classified as physically inactive. **Conclusions:** This study revealed that most patients on HD in Saudi Arabia have positive perceptions regarding the benefits of exercise. Nevertheless, several barriers were also reported by these patients, with tiredness, worry about affecting the arteriovenous fistula, body pain, and muscle fatigue being the most reported barriers. Further studies are necessary to investigate the relationship between a positive perception of exercise benefits and exercise engagement.

## 1. Introduction

Chronic kidney disease (CKD) is a chronic condition [1] and is a primary global health concern [1,2,3]. The majority of CKD cases ultimately progress to end-stage renal disease (ESRD), an irreversible loss of kidney function (kidney failure) [4,5], which requires treatment by renal replacement therapy (RRT), including renal transplantation, hemodialysis (HD), or peritoneal dialysis [6]. The literature estimates that approximately 4.9 to 9.7 million patients with ESRD need RRT globally [7]. Moreover, HD is the principal RRT modality for patients with ESRD. Those patients on HD treatment require connection to an HD machine two or three times per week, each time taking about 4 h [8,9]. Thus, it consumes patients’ time; consequently, patients remain physically inactive for an extended period [9], which results in a decline in independence and mobility as well as acceleration in physical deterioration [10,11]. Research has shown an association between low levels of physical exercise and high mortality, poor body function, and increased symptom burden in dialysis patients [12,13]. On the other hand, earlier studies and guidelines highlight the significance and benefits of physical exercise for patients on HD [14,15,16,17]. A recent umbrella review of 44 meta-analyses on 35,432 patients with CKD found that physical exercise is a potential non-pharmacological therapy [18]. It effectively eliminates or controls several complications of dialysis [19]. Additionally, research has reported improvements in muscle function and a reduction in mortality, cardiovascular instability, and muscle cramps in patients on HD who engage in higher levels of physical exercise [20]. Despite these benefits, patients on HD reported lower levels of physical exercise than the general public [21].

A previous study by Alshehri M.A. et al. in 2025 reported that the prevalence of CKD in Saudi Arabia is around 4.8% [22]. Another study by the Saudi Center for Organ Transplantation in 2019 reported that the total number of patients with ESRD is around 28,256, with an estimated prevalence rate of 0.09% [23]. A previous systematic review in Saudi Arabia reported that the majority of Saudi Arabians are not active enough to meet the recommended guidelines for physical activity, specifically females [24]. This systematic review identified that extreme weather (daytime temperature and high solar radiation), cultural barriers, urbanization (car-dependent lifestyle), heavy traffic, lack of social support, and lack of time are the main barriers towards performing physical activities in Saudi Arabia [24]. Exercise and physical activity culture in Saudi Arabia is limited due to several reasons, mainly due to the climate and the growing urbanization environment [24]. The reduced female physical activity in Saudi Arabia could be attributed mainly to social, cultural and traditional barriers, as females have limited opportunities to engage in physical activity, especially outdoor leisure-type activity [24].

A previous study in Saudi Arabia found that there is a critical gap in the availability of exercise programs for patients with CKD [25]. Moreover, the majority of physicians acknowledged the role of exercise in patients with CKD and advised them regarding physical activity on a frequent basis [25]. Evaluating the perception of benefits and barriers to exercise for patients on HD is crucial in identifying tailored exercise interventions that are proper for these patients [26]. The perception of benefits and barriers of exercise among patients on HD is linked with their exercise behavior. The high perception of barriers to exercise may discourage patients from engaging in physical exercise, while the high perception of the benefits of exercise can encourage them [27]. However, the perception of benefits and barriers to exercise for patients on HD is affected by cultural factors [28]. There are limited studies that examined patients on HD who perceived exercise benefits and barriers in Saudi Arabia. Considering the unique culture and high prevalence of physical inactivity among the population in Saudi Arabia [24,29], such a study is required in this area. Therefore, this study aims to investigate perceptions of exercise benefits and barriers among patients on HD in Saudi Arabia.

This study hypothesized that the sociodemographic characteristics of patients on HD might affect their perceptions of the benefits and barriers of physical exercise.

## 2. Methods

### 2.1. Study Design and Settings

This is a cross-sectional survey study that was conducted in two dialysis centers (Dr. Sulaiman Al Habib Hospital in Riyadh and Abdulkarim Bakr Medical center in Jeddah) in Saudi Arabia between May and September 2025.

### 2.2. Study Population and Sampling Procedure

Patients undergoing HD formed the study population. Patients were recruited using the convenience sampling technique. The inclusion criteria were patients aged ≥ 18 years, diagnosed with ESRD and receiving maintenance in-center hemodialysis, on hemodialysis for at least 3 months before enrollment, clinically stable at the time of survey administration, able to provide informed consent, and able to understand and complete the questionnaire (self-administered or interviewer-administered). The exclusion criteria were patients who are unable to provide informed consent (e.g., significant cognitive impairment or delirium), those who are currently hospitalized or acutely medically unstable (e.g., acute coronary syndrome, decompensated heart failure, sepsis), those who have severe psychiatric illness or cognitive disorder interfering with reliable questionnaire completion, and those who have major communication barriers that prevent questionnaire completion despite assistance (e.g., severe aphasia, severe uncorrected visual or hearing impairment, language barrier).

### 2.3. Study Instrument

In this research, the Dialysis Patient-perceived Exercise Benefits and Barriers Scale (DPEBBS) was used to examine patients’ perceived benefits and barriers to exercise [27]. This scale utilized a 4-point Likert scale to rate patients’ answers that ranged from ‘1’ for ‘strongly disagree’ to ‘4’ for ‘strongly agree’. The mean score was estimated by summing all respondents’ numerical responses for each item, divided by the total number of participants. In the original questionnaire tool by Zheng et al., the DPEBBS items were categorized into two domains: the perceived benefits (12 items) and perceived barriers to exercise (12 items). The categorization was performed during the questionnaire development. The second section examined patients’ physical activity status, which was evaluated using the ‘General Practice Physical Activity Questionnaire’ (GPPAQ) [30]. The GPPAQ classified the physical activity of the patients into four categories: active, moderately active, moderately inactive, and inactive.

The questionnaire tools were translated into the Arabic language using the forward-backward translation technique by two independent reviewers. The translation is conceptual-focused rather than word-by-word translation. The translated version was piloted on a small number of the targeted study population. The study participants in the piloting phase confirmed that the questionnaire items are clear and easy to understand.

### 2.4. Ethical Approval

This study was approved by the Bioethics Committee of Scientific and Medical Research at the University of Jeddah, Jeddah, Saudi Arabia (UJ-REC-299). All participants provided their consent before participation in the study. Permission was obtained from the original developers for the use of both the DPEBBS and the GPPAQ.

### 2.5. Data Analysis

**Figure 1:**
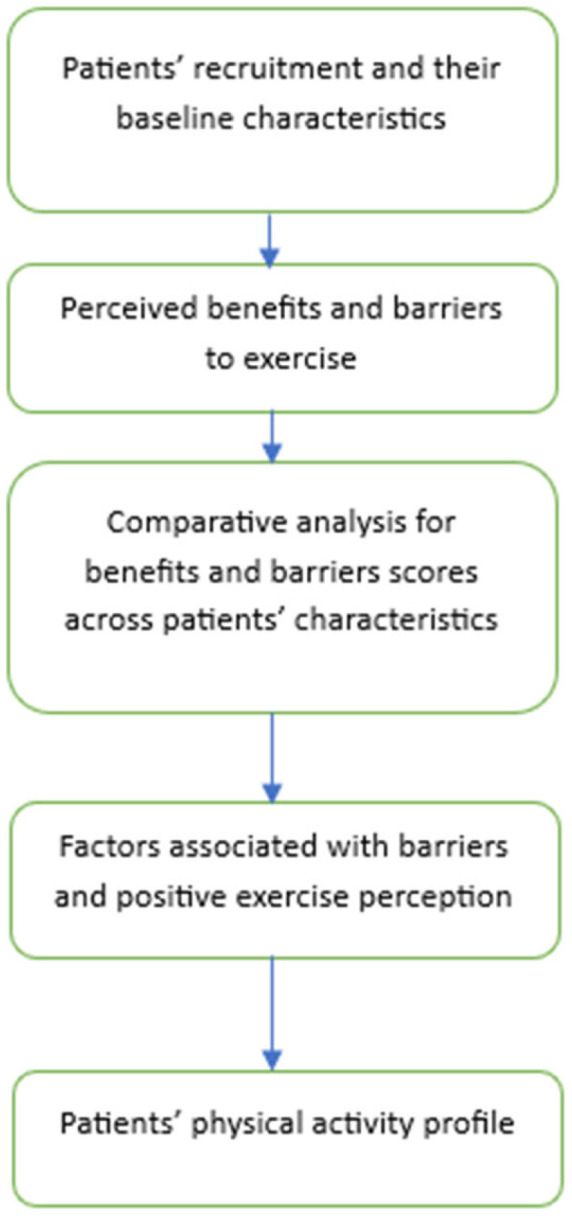
Analytical flowchart for the research.

Descriptive statistics were utilized to summarize the demographic variables. Frequency and percentage were used to report categorical variables, while mean and standard deviation (SD) were used for continuous data such as the benefits and barrier scores. The Kolmogorov–Smirnov test of normality was applied, and since the data supported parametric assumptions, the Analysis of Variance (ANOVA) test and the independent *t*-test were performed when applicable. For multiple group comparisons, the Tukey post hoc test was applied, and the results were evaluated accordingly. The internal consistency was evaluated by Cronbach’s alpha, with values >0.7 considered acceptable. To identify factors associated with benefits and barriers, the scores were categorized based on their median values. For benefits and barriers, the median was 38 and 29, respectively. A multiple logistic regression was performed to assess the factors associated with benefits and barriers as a dependent variable separately. The results from the multivariate regression analyses are presented as adjusted odds ratios (aOR) with 95% confidence intervals (CI) and corresponding *p*-values. All barrier items were reverse-coded before analysis. The level of significance was defined as α = 0.05. All calculations and analyses were carried out with the SPSS (Statistical Package of Social Sciences Version 28.0) program. Figure 1 below presents the step-by-step analysis process for this research.

### 2.6. Reliability and Internal Consistency Testing for DPEBBS

The internal consistency for benefits and barriers was assessed by Cronbach’s alpha. For benefits, it was 0.92 and 0.86 for barriers, which indicates a high internal consistency.

### 2.7. Sample Size

This is an exploratory study with no formal a priori sample size calculation. Therefore, all patients who met the inclusion criteria and agreed to participate were included in this research.

## 3. Results

### 3.1. Sociodemographic Characteristics

This study included 104 patients with ESRD, of whom 61 (58.7%) were male, and 43 (41.3%) were female. Most participants were non-Saudi (72, 69.2%), while 32 (30.8) were Saudi. Regarding marital status, 68 (65.4%) were married, and 18 (17.3%) were single. In terms of education, the majority had a high school degree or less (50, 48.1%), followed by 37 participants with a bachelor’s degree (35.6%). The most common occupations were housewives (30, 28.8%) and retired individuals (27, 26.0%). The leading causes of ESRD were diabetes mellitus and hypertension (36, 34.6%) for both. Further details regarding the participants’ demographics are provided in Table 1.

Table 2 shows that among the included patients with ESRD, hypertension was the most prevalent comorbidity, reported in 89 (85.6%) individuals. Diabetes mellitus was also common, affecting 44 (42.3%) patients, Table 2.

### 3.2. Perceived Benefits

Table 3 shows that most patients expressed positive perceptions regarding the benefits of exercise. The majority agreed or strongly agreed that exercise improves mood (91, 87.5%) with a mean agreement score of 3.4 (SD: 0.7), prevents muscular atrophy (85, 81.8%), postpones decline in body function (85, 81.7%), and enhances quality of life (87, 83.7%). A similarly high proportion believed that exercise helps maintain steady body weight (87, 83.7%) and improves self-care abilities (83, 79.8%). In contrast, fewer participants strongly endorsed the idea that exercise reduces medical costs or prevents other diseases, with a mean agreement score of 2.8 (SD: 0.9). More detailed distributions of responses for each item are provided in Table 3.

### 3.3. Perceived Barriers

Regarding barriers to exercise, several concerns were frequently reported by patients. Table 4 shows that the most prominent barrier was worry about affecting the arteriovenous fistula, with 71 (68.2%) participants strongly agreeing or agreeing with this concern, with a mean agreement score of 3.1 (SD: 1.0). Tiredness (73, 70.2%) and muscle fatigue (66, 63.5%) were also commonly cited obstacles, with a mean score of 3.1 (SD: 1.0) and 3.0 (SD: 1.0), respectively. Fear of falling was reported by 60 (57.7%) of participants, while body pain was reported as a barrier by 67 (64.4%), Table 4.

The mean of perceived benefits score was 37.3 ± SD: 7.24, and for barriers was 27.44 ± SD: 7.32. Table 5 shows that male participants reported higher perceived benefits than females (38.93 ± SD: 6.06) and (35.00 ± SD: 8.18), respectively (p = 0.006). Regarding marital status, single individuals had higher benefits scores (40.72 ± SD: 5.03) compared to married (37.34 ± SD: 6.25 and other groups (33.78 ± SD: 10.66) (*p* = 0.015). For barriers, Table 5 shows that male participants reported a higher barrier score than females (28.67 ± SD: 6.92) and (25.70 ± SD: 7.61), (*p* = 0.04), respectively. Participants with higher education levels had a significantly greater barrier score (*p* = 0.005), Table 5.

In the multiple logistic regression analysis assessing factors associated with perceived benefits of exercise, older age was significantly associated with lower odds of perceiving exercise as beneficial (aOR = 0.94; 95% CI: 0.91–0.98; *p* = 0.008). This indicates that elderly patients were slightly less likely to report positive perception towards exercise, Table 6.

In the multiple logistic regression model assessing factors associated with perceived barriers to exercise, having a university degree was associated with higher odds of reporting exercise barriers compared with having no education (aOR = 16.22, 95%:1.29–204.42, *p* = 0.03), Table 7.

### 3.4. Physical Activities Profile

Based on the GPPAQ physical activity classification criteria, 78.8% of the patients (n = 82) are classified as physically inactive, 15.4% (n = 16) moderately inactive, 3.8% (n = 4) moderately active, and 1.9% (n = 2) active. Table 8 and Table 9 present the type and amount of physical activity involved in patients’ work and the time spent on physical activities.

When the patients were asked about how they would describe their usual walking pace, 63.5% of the patients described it as slow pace “less than 3 mph”, 33.7% as steady average pace, and 2.9% as fast pace “over 4 mph”.

## 4. Discussion

Most patients on HD in the current study expressed positive perceptions regarding the benefits of exercise. Consistent with our findings, previous investigations among dialysis patients in the US have shown that most patients perceive exercise as beneficial [31,32]. Likewise, in Australia [9], most patients with ESRD who receive HD perceived exercise as positive; most of them agreed that exercise improves mood and QoL, prevents muscular wasting, postpones decline in body function, and provides other benefits. Similarly, prior research indicated that patients with ESRD who receive HD in Jordan [33] and patients on HD in Turkey [34] perceived significantly higher exercise benefits than exercise barriers. Furthermore, the most commonly reported exercise benefits in those two prior studies were improved mood and prevention of muscular atrophy in the Jordan study [33], and improved QoL and prevention of muscular atrophy in the Turkey study [34]. These findings indicate that patients on HD had favorable attitudes toward incorporating physical exercise into their treatment. Indeed, earlier investigations conducted in Saudi Arabia [35] and other countries [36] have established the psychological and physical benefits of exercise for patients on HD treatment. However, other studies found a moderate positive perception regarding the benefits of exercise among patients on HD [26,37]. The small differences between studies could be due to discrepancies in factors such as participants’ culture, demographics, level of physical activity, and exercise knowledge. For instance, prior research documented higher perceived psychological benefits of exercise among dialysis patients with higher physical activity levels [38]. Hence, individualized exercise interventions that consider patients’ factors may increase positive perceptions about the benefits of exercise.

Still, the high positive perception of exercise benefits among patients does not necessarily mean that they will engage in exercise. A prior investigation among patients on HD in Iran revealed that, due to the exercise barrier, the majority of the participants do not engage in exercise, despite having a high positive perception of exercise [28]. In addition, as the perception of obstacles increased, the engagement in exercise decreased [27]. In this study, several barriers were frequently reported by patients on HD, with tiredness (70.2%), worry about affecting the arteriovenous fistula (68.2%), body pain (64.4%), and muscle fatigue (63.5%) being the most commonly cited barriers. These findings align with some previous studies and differ from others. For instance, in Australia [9], muscle fatigue, body pain, and tiredness were the most frequently reported barriers among patients with ESRD who received HD; however, only 20% of these patients expressed concern about the arteriovenous fistula. Moreover, lower-extremity fatigue and tiredness were the leading barriers in Jordan [33]. Fatigue and thirst were the most frequent barriers in Turkey [34]. In China, a lack of exercise knowledge, muscle fatigue, and tiredness were the predominant barriers [39,40]. Another prior study conducted among patients on HD in China identified fear of fistula injury, body pain, and fatigue as the primary barriers to exercise [27]. Fear related to arteriovenous fistula, lower-extremity muscle fatigue, and tiredness were the most common barriers to exercise in Iran [28]. A prior study in the UK also documented disease-specific barriers (such as fatigue) and general barriers (such as pain and fear of falls) among patients on HD [21]. Other research has found that concerns related to arteriovenous fistula were the leading barrier to exercise [32,41]. Indeed, physical activity barriers vary among patients on HD based on factors related to patients (socioeconomic status, cultural, psychological, and physical) and to disease (such as comorbidities) [42], besides differences in treatment and clinical course for each patient, these may explain the variety in reported barriers between studies [27]. Therefore, tailored exercise interventions based on each case could aid in overcoming exercise obstacles and thereby encourage patients to follow the doctor’s recommendations regarding exercise.

Indeed, the literature underscores that patients with ESRD experienced significant arterial hypotension, declined exercise tolerance, nutritional deficiencies, muscle wasting, and sarcopenia during HD sessions, and they experienced fatigue post-dialysis, which results in sedentary behaviors among those patients [43,44]. Post-dialysis fatigue and decline in physical function may contribute to muscle fatigue and tiredness among patients, and they can overcome these difficulties through physical exercise [45,46]. Research demonstrated multiple motivations for exercise in patients on HD, including educational interventions, tailored exercise programs [21], and awareness of exercise benefits [47]. Hence, such interventions may increase engagement in exercise.

This study showed that the age of participants was associated with significantly lower odds of perceiving exercise as beneficial, indicating a slightly lower likelihood of documenting a positive perception regarding exercise among patients for each additional year of patients’ age. In line with these, a prior study among Brazilian patients on HD revealed a significantly lower benefit score for exercise among older patients compared to younger patients [48]. In fact, the variation in the effect of exercise on patients on HD at advanced age versus middle age has been established across previous studies [49]. The significant decrease in older patients on HD perception of the benefits of exercise may be attributed to several factors. Firstly, earlier research underlines that older adults may encounter challenges, such as a lack of confidence and fear of injury [29]. Secondly, multiple studies found that symptom load was higher among older dialysis patients [50,51], which was also associated with higher comorbidities [35,52,53,54]. Consistent with this, worries about HD symptoms among patients with ESRD who are on HD in Australia increased with age [9]. Additionally, a prior investigation conducted to assess barriers to exercise among adult individuals in Saudi Arabia noted that external and internal exercise barriers increased among older people [29]. Finally, other studies revealed that physical activity declined among older people due to limited mobility, health issues, and diminished motivation [55,56]. Thus, age-specific exercise interventions are required.

One interesting observation in our study is that a significant association exists between higher odds of reporting exercise barriers and patients with a bachelor’s degree compared with those with no education. Our findings may reflect the limited time and sedentary nature of jobs for individuals with higher education, which can increase perceived barriers to exercise in rapidly urbanizing nations, such as Saudi Arabia [29,57,58,59,60,61]. Consistent with this, research reported that urbanization contributes to low levels of physical exercise among the inhabitants [62]. On the contrary, other studies suggest that individuals with higher educational levels, including those with HD, have better knowledge, information, and awareness of health and healthier lifestyles [63,64]. Likewise, another research, among patients with chronic diseases, found better health literacy among patients with higher education [65]. Accordingly, these prior studies indicated that patients with higher education were more likely to participate in exercise and understand its benefits, subsequently having lower odds of reporting exercise barriers. This contrast may be related to differences in cultural, environmental, and socioeconomic factors. Culturally sensitive education campaigns, workplace interventions, and improving access to exercise facilities are recommended [29], with a focus on individuals who have a bachelor’s degree.

Healthcare professionals should balance the benefits of physical exercise with kidney-related risks, promoting safe physical exercise practices to optimize renal outcomes and the overall health of patients with ESRD [66]. Based on the findings of this research, healthcare professionals are advised to focus on modifiable barriers related to performing physical activities, such as tiredness, muscle fatigue and fear of arteriovenous fistula damage. Healthcare professionals should provide education and increase patients’ awareness regarding the importance of physical activity and correct any misunderstood information. Educational initiatives should consider patients’ age and literacy levels.

This study has several strengths. This study is among the first few studies that examined patients on HD perceived exercise benefits and barriers in Saudi Arabia. Furthermore, this study utilized previously developed and validated questionnaire tools, which increase the reliability of the study findings. Moreover, all study participants were recruited from HD units, and their diagnosis is medically confirmed, reducing misclassification bias. On the other hand, this study has limitations. The cross-sectional study design has a restricted ability to examine causality across the study variables. Furthermore, the utilization of the convenience sampling technique decreases the generalizability of the study findings. Moreover, the utilization of self-reported measures is prone to recall bias and social desirability bias. In addition, the DPEBBS has known limitations regarding internal consistency and factor structure (specifically the imbalance where Factor 1 contains 10 items while others contain only 2–4). Therefore, the study findings should be interpreted carefully.

## 5. Conclusions

This study revealed that most patients on HD in Saudi Arabia have positive perceptions regarding the benefits of exercise. Nevertheless, several barriers were also reported by these patients, with tiredness, worry about affecting the arteriovenous fistula, body pain, and muscle fatigue being the most reported barriers. Tailored exercise interventions may enhance positive perceptions of exercise benefits and help in overcoming exercise barriers. The age of participants was associated with significantly lower odds of perceiving exercise as beneficial, while a bachelor’s degree was associated with significantly higher odds of perceiving exercise barriers. Therefore, exercise programs should be age-specific and education-tailored for elderly patients and other patients with low education level to enhance patients’ awareness and improve their attitude towards physical activity. Decision-makers are advised to integrate physical exercise in routine dialysis care protocols. Further longitudinal multicenter studies are necessary to investigate the relationships between a positive perception of exercise benefits and exercise engagement. Furthermore, future studies should adopt a random sampling technique to enhance the generalizability of the study findings.

## Figures and Tables

**Table 1:** Sociodemographic characteristics of ESRD patients.

Sociodemographic Characteristics		N	%
Gender	Male	61	58.7%
	Female	43	41.3%
Nationality	Non-Saudi	72	69.2%
	Saudi	32	30.8%
Marital Status	Single	18	17.3%
	Married	68	65.4%
	Other	18	17.3%
Highest education	No education	12	11.5%
	High school or less	50	48.1%
	Bachelor	37	35.6%
	Master’s or PhD	5	4.8%
Occupation	Non employed	8	7.7%
	Self-employed	8	7.7%
	Student	3	2.9%
	Employee in the private sector	17	16.3%
	Housewife	30	28.8%
	Retired	27	26.0%
	Other	4	3.8%
	Employee in the governmental sector	7	6.7%
Hemodialysis Center	Dr. Sulaiman Alhabib	34	32.7%
	Abdulkarim Bakr Medical Center	70	67.3%
The cause of ESRD	Diabetes mellitus	36	34.6%
	Hypertension	36	34.6%
	Hereditary Kidney Disease	8	7.7%
	Glomerulonephritis	10	9.6%
	Unknown	13	12.5%
	None	1	1.0%

**Table 2:** Comorbidities among included patients with ESRD.

Comorbidities History	N	%
Diabetes Mellitus	44	42.3%
Hypertension	89	85.6%
Cardiac disease	17	16.3%
Cerebrovascular Accident	5	4.8%
Chronic lung disease	7	6.7%

**Table 3:** Perceived benefits of exercise among patients with ESRD.

	Strongly Disagree	Disagree	Agree	Strongly Agree	The Mean Score (Standard Deviation)
Exercise helps reduce my total medical costs	7 (6.7%)	28 (26.9%)	35 (33.7%)	34 (32.7%)	2.9 (0.9)
Exercise helps reduce my body pain	4 (3.8%)	23 (22.1%)	37 (35.6%)	40 (38.5%)	3.1 (0.9)
Exercise can postpone a decline in body function	4 (3.8%)	15 (14.4%)	41 (39.4%)	44 (42.3%)	3.2 (0.8)
Exercise prevents muscular atrophy	4 (3.8%)	15 (14.4%)	37 (35.6%)	48 (46.2%)	3.2 (0.8)
Exercise improves my mood	1 (1.0%)	12 (11.5%)	41 (39.4%)	50 (48.1%)	3.4 (0.7)
Exercise improves bone diseases	2 (1.9%)	23 (22.1%)	43 (41.3%)	36 (34.6%)	3.1 (0.8)
Exercise improves my appetite	2 (1.9%)	25 (24.0%)	51 (49.0%)	26 (25.0%)	3.0 (0.8)
Exercise helps me lead an optimistic and active life	3 (2.9%)	14 (13.5%)	44 (42.3%)	43 (41.3%)	3.2 (0.8)
Exercise improves my quality of life	1 (1.0%)	16 (15.4%)	45 (43.3%)	42 (40.4%)	3.2 (0.7)
Exercise can keep my body weight at a steady level	2 (1.9%)	15 (14.4%)	58 (55.8%)	29 (27.9%)	3.1 (0.7)
Exercise helps enhance my self-care abilities	3 (2.9%)	18 (17.3%)	44 (42.3%)	39 (37.5%)	3.1 (0.8)
Exercise will keep me from having other diseases (e.g., cold)	11 (10.6%)	28 (26.9%)	40 (38.5%)	25 (24.0%)	2.8 (0.9)

**Table 4:** Perceived barriers to exercise among patients with ESRD.

	Strongly Agree	Agree	Disagree	Strongly Disagree	The Mean Score (Standard Deviation)
Tiredness	45 (43.3%)	28 (26.9%)	30 (28.8%)	1 (1.0%)	3.1 (0.9)
Adverse to health	16 (15.4%)	11 (10.6%)	64 (61.5%)	13 (12.5%)	2.3 (0.9)
Fear of falling	33 (31.7%)	27 (26.0%)	35 (33.7%)	9 (8.7%)	2.8 (1.0)
Muscle fatigue	39 (37.5%)	27 (26.0%)	32 (30.8%)	6 (5.8%)	3.0 (1.0)
Lack of understanding of benefits	14 (13.5%)	22 (21.2%)	60 (57.7%)	8 (7.7%)	2.4 (0.8)
Other comorbidities	34 (32.7%)	16 (15.4%)	42 (40.4%)	12 (11.5%)	2.7 (1.1)
Body pain	34 (32.7%)	33 (31.7%)	31 (29.8%)	6 (5.8%)	2.9 (0.9)
Lack of exercise knowledge	18 (17.3%)	17 (16.3%)	58 (55.8%)	11 (10.6%)	2.4 (0.9)
Worry about thirst	20 (19.2%)	29 (27.9%)	46 (44.2%)	9 (8.7%)	2.6 (0.9)
Have chronic kidney disease	30 (28.8%)	15 (14.4%)	45 (43.3%)	14 (13.5%)	2.6 (1.0)
Worry about arteriovenous fistula	51 (49.0%)	20 (19.2%)	24 (23.1%)	9 (8.7%)	3.1 (1.0)
Burden on family	34 (32.7%)	20 (19.2%)	37 (35.6%)	13 (12.5%)	2.7 (1.1)

**Table 5:** Comparison of benefits and barriers scores across sociodemographic characteristics.

Variable		Benefits		Barriers	
		Mean ± SD	*p* Value	Mean ± SD	*p* Value
Gender (n = 104)	Male	38.93 ± 6.06	0.006	28.67 ± 6.92	0.040
	Female	35.00 ± 8.18		25.70 ± 7.61	
Nationality (n = 104)	Non-Saudi	36.69 ± 7.96	0.190	26.81 ± 7.94	0.180
	Saudi	38.69 ± 5.13		28.88 ± 5.55	
Marital Status (n = 104)	Single	40.72 ± 5.03	0.015	29.33 ± 9.93	0.262
	Married	37.34 ± 6.25		27.50 ± 6.60	
	Other	33.78 ± 10.66		25.33 ± 6.81	
Highest education (n = 104)	No education	30.50 ± 7.34	<0.001	20.50 ± 5.87	0.005
	High school or less	37.38 ± 7.63		28.22 ± 7.48	
	Bachelor	38.49 ± 5.42		28.38 ± 6.47	
	Master ot PhD	44.20 ± 3.90		29.40 ± 7.80	
Occupation (n = 104)	Non employed	36.50 ± 7.98	0.002	29.75 ± 4.06	0.011
	Self-employed	43.63 ± 4.90		30.75 ± 11.21	
	Student	43.00 ± 2.65		38.33 ± 4.04	
	Employee in the private sector	38.41 ± 4.77		30.12 ± 6.69	
	Housewife	33.87 ± 8.35		24.23 ± 6.70	
	Retired	37.81 ± 6.43		26.70 ± 5.61	
	Other	31.25 ± 6.34		26.25 ± 9.03	
	Employee in the governmental sector	42.14 ± 3.72		27.14 ± 8.93	
Hemodialysis Center (n = 104)	Dr. Sulaiman Alhabib	38.44 ± 5.11	0.268	28.71 ± 5.36	0.222
	Abdulkarim Bakr Medical Center	36.76 ± 8.06		26.83 ± 8.07	
The cause of ESRD (n = 104)	Diabetes mellitus	35.31 ± 8.15	0.165	26.00 ± 7.53	0.604
	Hypertension	37.58 ± 6.69		27.83 ± 7.42	
	Hereditary Kidney Disease	41.63 ± 3.42		28.00 ± 6.85	
	Glomerulonephritis	37.60 ± 9.24		29.90 ± 4.43	
	Unknown	38.77 ± 5.07		27.54 ± 8.71	

ESRD: End-stage renal disease. The ANOVA test and the independent *t*-test were performed when applicable. For multiple group comparisons, the Tukey post hoc test was applied.

**Table 6:** Factors associated with positive perceptions toward exercise.

Variables		aOR (95% Confidence Interval) *	*p*-Value
	Age in years	0.94 (0.91–0.98)	0.008
Gender	Male	Reference	
	Female	0.26 (0.05–1.44)	0.123
Nationality	Non-Saudi	Reference	
	Saudi	1.34 (0.42–4.34)	0.623
Marital status	Single	Reference	0.278
	Married	0.90 (0.16–4.93)	0.901
	Other	3.02 (0.33–27.97)	0.330
Years on dialysis	Less than a year	Reference	0.909
	1–3	1.32 (0.31–5.65)	0.711
	>3 years	1.37 (0.33–5.68)	0.669
Education level	No education	Reference	0.217
	High school or less	3.20 (0.53–19.20)	0.204
	University degree	5.28 (0.79–35.29)	0.086
Occupation	Not employed, self-employed, students	Reference	0.061
	Private	0.15 (0.03–0.78)	0.025
	Housewife	0.56 (0.07–4.21)	0.572
	Retired and governmental employees	1.00 (0.21–4.86)	0.997
Causes of ESRD	DM	Reference	0.984
	HTN	1.12 (0.35–3.56)	0.850
	Hereditary Kidney Disease and other causes	0.94 (0.21–4.14)	0.937

aOR: Adjusted odds ratio, ESRD: End stage renal disease, * Based on their clinical relevance and literature review all of the following factors were included as covariates in the regression model (gender, nationality, marital status, years on dialysis, education level, occupation, and causes of ESRD), the multiple logistic regression analysis was applied to estimate the aOR.

**Table 7:** Factors associated with barriers toward exercise.

Variables		aOR (95%CI)	*p* Value
Age in years		0.97 (0.93–1.01)	0.137
Gender	Male	Reference	
	Female	0.50 (0.09–2.76)	0.423
Nationality	Non-Saudi	Reference	
	Saudi	2.24 (0.68–7.37)	0.184
Marital status	Single	Reference	
	Married	2.93 (0.53–16.18)	0.219
	Other	3.07 (0.33–29.00)	0.327
Years on dialysis	Less than a year	Reference	
	1–3	0.43 (0.09–1.97)	0.279
	>3 years	0.34 (0.08–1.48)	0.149
Education level	No education	Reference	
	High school or less	8.96 (0.76–106.26)	0.082
	University degree	16.22 (1.29–204.42)	0.031
Occupation	Not employed, self-employed, students	Reference	
	Private	0.23 (0.04–1.30)	0.096
	Housewife	0.13 (0.02–1.16)	0.067
	Retired and governmental employees	0.15 (0.03–0.78)	0.024
Causes of ESRD	DM	Reference	
	HTN	2.66 (0.74–9.57)	0.134
	Hereditary Kidney Disease and other causes	0.63 (0.15–2.72)	0.536
	Glomerulonephritis	3.54 (0.51–24.36)	0.199

aOR: Adjusted odds ratio; ESRD: End-stage renal disease. The multiple logistic regression analysis was applied to estimate the aOR. Based on their clinical relevance and literature review, all of the following factors were included as covariates in the regression model (gender, nationality, marital status, years on dialysis, education level, occupation, and causes of ESRD).

**Table 8:** Type and amount of physical activity involved in patients’ work.

General Practice Physical Activity Questionnaire Items		N (%)
A	I am not in employment (retired, retired for health reasons, unemployed, full-time career, etc.)	75 (72.1%)
B	I spend most of my time at work sitting (such as in an office)	22 (21.2%)
C	I spend most of my time at work standing or walking. However, my work does not require much intense physical effort (e.g., shop assistant, hairdresser, security guard, childminder, etc.)	6 (5.8%)
D	My work involves definite physical effort, including handling of heavy objects and use of tools (e.g., plumber, electrician, carpenter, cleaner, hospital nurse, gardener, postal delivery workers, etc.)	1 (1.0%)
E	My work involves vigorous physical activity, including handling of very heavy objects (e.g., scaffolder, construction worker, refuse collector, etc.)	0 (0%)

**Table 9:** Time spent on physical activities.

During Last Week, How Many Hours Did You Spend on Each of the Following Activities?		None	Some but Less Than 1 h	1 h but Less Than 3 h	3 h or More
A	Physical exercise such as swimming, jogging, aerobics, football, tennis, gym, workout, etc.	90 (86.5%)	10 (9.6%)	2 (1.9%)	2 (1.9%)
B	Cycling, including cycling to work and during leisure time	99 (95.2%)	4 (3.8%)	1 (1.0%)	0 (0%)
C	Walking, including walking to work, shopping, for pleasure, etc.	34 (32.7%)	39 (37.5%)	28 (26.9%)	3 (2.9%)
D	Housework/Childcare	65 (62.5%)	21 (20.2%)	16 (15.4%)	2 (1.9%)
E	Gardening/DIY	86 (82.7%)	15 (14.4%)	2 (1.9%)	1 (1.0%)

## Data Availability

The raw data supporting the conclusions of this article will be made available by the authors on request.

## Abbreviations

CKD	Chronic kidney disease
ESRD	End-stage renal disease
RRT	Renal replacement therapy
HD	Hemodialysis
DPEBBS	Dialysis Patient-perceived Exercise Benefits and Barriers Scale
GPPAQ	General Practice Physical Activity Questionnaire
SD	Standard deviation
ANOVA	Analysis of Variance
aOR	Adjusted odds ratios
CI	Confidence intervals
SPSS	Statistical Package of Social Sciences Version
